# Oncovascular Resection in Pelvic Exenteration: A Systematic Review

**DOI:** 10.1111/ans.70824

**Published:** 2026-07-07

**Authors:** Joshua Richard Burke, Yasmin Oskui, Imogen Samuel, Jonathan Wild, Kilian G. Brown, Jonathan Ghosh, Paul A. Sutton

**Affiliations:** ^1^ Colorectal & Peritoneal Oncology Centre The Christie NHS Foundation Trust Manchester UK; ^2^ Division of Cancer Sciences, Manchester Cancer Research Centre The University of Manchester Manchester UK; ^3^ Manchester Vascular Centre Manchester University NHS Foundation Trust Manchester UK; ^4^ Department of Colorectal Surgery and Surgical Outcomes Research Centre Royal Prince Alfred Hospital Sydney Australia

## Abstract

**Background:**

Resection of major vascular structures may be required to achieve R0 resection in pelvic exenteration surgery for advanced pelvic malignancy. The evidence base for pelvic oncovascular techniques is limited, with patient outcomes following these procedures not widely reported.

**Objectives:**

This systematic review synthesises the current literature on R0 resection and 30‐day mortality rates following oncovascular pelvic exenteration.

**Methods:**

A systematic review was conducted according to a pre‐defined protocol (PROSPERO ID CRD42024613055). MEDLINE, EMBASE, Cochrane Library and Web of Science were interrogated between January 1, 1947, to July 1, 2025. Studies exploring oncovascular resection in advanced pelvic malignancy were considered. Pre‐1980 studies were included narratively but excluded from quantitative synthesis. Data were extracted on R0 resection, postoperative morbidity and mortality. The ROBINS‐1 V2 tool was used to assess bias.

**Results:**

Seventeen studies met the inclusion criteria. After excluding three pre‐1980 studies, 411 contemporary patients were included in quantitative analysis. Among curative‐intent procedures (*n* = 334), the R0 resection rate was 65.3% (218/334). With regards to morbidity, Clavien–Dindo grade I–II complications occurred in 56.9% (227/399) and grade III–IV complications in 28.5% (109/382). Thirty‐day mortality was 1.9% (8/411). Median overall survival was 41 months (range 34–57.6), with median disease‐free survival of 27 months (range 8–43.2).

**Conclusion:**

Approximately two thirds of patients undergoing oncovascular pelvic exenteration achieved R0 resection, with morbidity and 30‐day mortality of 1.9%. Outcomes remain inferior to contemporary benchmark series for conventional exenteration, reflecting the pioneering phase of oncovascular practice. Centralisation, rigorous audit and benchmarking and improved patient selection should be the focus of future work to improve R0 and complication rates.

## Graphical abstract

Risk of bias assessment using ROBINS‐1 V2. Selection of the reported result (D7) was judged based on omission of key expected outcomes, including margin status (R0 resection), postoperative complications and mortality. Studies not reporting these outcomes were classified as serious risk of bias, while those with broadly complete but non‐pre‐specified reporting were classified as moderate. Created using robvis tool.

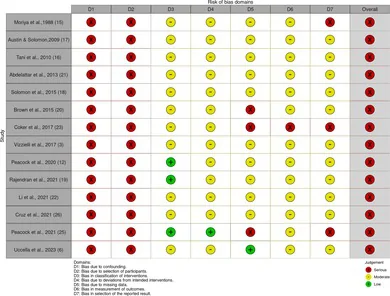

## 1 Introduction

In locally advanced pelvic malignancy, radical multi‐visceral resection in the form of pelvic exenteration has been used since the mid 20th century. Early attempts were associated with significant risk and cases involving major pelvic blood vessels were associated with high post‐operative mortality [1, 2]. Subsequently, the presence of invasion into the aorto‐iliac axis was considered a poor predictive indicator [1, 2]. Barber and Brunschwig remarked that the ‘permeation of disease to vessels at the peripheries of the pelvis carries a hopeless prognosis’ [1]. In the subsequent decades, vascular involvement was considered a relative contra‐indication to resection [1]. Zeitgeist has evolved; presently, ultra‐radical pelvic exenteration is reserved for attempts at R0 resection in advanced and recurrent cancers, due to the associated post‐operative physical and psychological morbidity [3]. The observed improvements in outcomes can be explained by advances in medical imaging and the incorporation of the multidisciplinary team approach [4]. When pelvic tumours invade the aorto‐iliac axis, ‘oncovascular’ surgery is performed, wherein vascular surgery techniques (i.e., vessel dissection, ligation, bypass and reconstruction) are employed to achieve tumour resection while maintaining pelvic and lower limb perfusion [5]. These approaches have also been utilised as a component of cytoreductive surgery in cases of peritoneal metastases [6].

When undertaking surgery for advanced pelvic malignancy, emphasis is placed on radical tumour excision and the approach to oncovascular resection in the pelvis is of interest across surgical oncology. Oncovascular pelvic surgery is precedented to show a rapid uptake, considering the increasing role of exenterative surgery [7, 8]. However, reporting of outcomes in the literature remains relatively limited. Invariably, heterogeneity exists in patient selection, operative approach and outcomes; a feature highlighted in a review of pelvic exenteration in the latest work by the United Kingdom Pelvic Exenteration Network (UKPEN) [8]. Rajendran et al. published a narrative synthesis outlining four of the key works within the field of oncovascular pelvic surgery. It concluded that although these ultra‐radical procedures are technically safe and feasible, there are limitations within the literature to inform best practice [7]. Furthermore, as oncovascular pelvic exenteration becomes commonplace in the treatment of locally recurrent disease, the operative field is set to be within a hostile abdomen which has undergone previous surgery and/or (re)irradiation, thereby making the complex procedure technically challenging. There is a need, therefore, to evidence the surgical approach and associated outcomes related to oncovascular pelvic exenteration, to inform best practice and to allow adequate shared decision making. To date, no secondary research has been published regarding outcomes following oncovascular pelvic exenteration. This systematic review aims to analyse the outcomes following pelvic tumour resection with oncovascular involvement, particularly R0 resection and 30‐day mortality. Secondary aims include obtaining values on long‐term survival and comprehensively outline the techniques utilised.

## 2 Methods

A systematic review examining pre‐determined outcomes following oncovascular resection in advanced pelvic malignancy was conducted and reported in accordance with an approved strategy from the Preferred Reporting Items for Systematic Reviews and Meta‐Analyses (PRISMA) guidelines [9]. This study was registered with PROSPERO on 26 November 2024 (CRD42024613055).

### 2.1 Inclusion Criteria

The literature is replete with papers reporting outcomes following major pelvic resection; however, outcomes following resection of tumours associated with the aorto‐iliac axis are limited. Therefore, the search was restricted to patients who had undergone pelvic exenteration combined with oncovascular resection ± reconstruction of the aorto‐iliac axis. To qualify for inclusion within this study, reports had to fulfil all the following criteria:

1. The procedure performed was pelvic exenteration, defined for the purpose of this study as multi‐visceral resection within the pelvis;
2. There was involvement of major pelvic vessel(s), defined as the aorta, inferior vena cava, common/internal/external iliac arteries and/or veins, overall termed the aorto‐iliac axis;
3. Oncovascular intervention was performed in pelvic exenteration, defined as en bloc tumour resection involving major pelvic vessels (aorto‐iliac axis), with vascular procedures including ligation and reconstruction (interposition grafting or bypass) and referred to as oncovascular resection (±reconstruction);
4. There was specification of which oncovascular techniques were performed including ligation, reconstruction and bypass;
5. There is reporting of outcomes within the group which received oncovascular intervention.

Primary research studies, including case series, cohort studies and randomised controlled trials, were included. Conference abstracts and case reports were excluded. Reports not available in the English language were excluded. Tumour histology was not restricted, as the review focused on operative approach (pelvic exenteration with oncovascular resection ± reconstruction) rather than tumour subtype.

Because surgical, anaesthetic and imaging practices prior to 1980 differ substantially from modern oncovascular standards, pre‐1980 studies were included in the qualitative synthesis but excluded from all pooled quantitative analyses. This approach aligns with best‐practice recommendations to minimise era‐related heterogeneity.

### 2.2 Search Strategy and Data Extraction

The OVID SP versions of MEDLINE (covering 1946 through 2025) and EMBASE (1947 through 2025), along with the Cochrane Library and the Web of Science, were searched using a customised search strategy (Table S2). The search strategy combined free‐text terms and controlled vocabulary related to vascular anatomy, surgical procedures and oncologic interventions. In Ovid MEDLINE, terms were restricted to title fields (.m_titl.), using truncation (*) and Boolean operators (AND/OR). The strategy was adapted appropriately for each database's syntax. The following terms were combined as follows:

1. Vascular anatomy terms: “arter*” OR “vein*” OR “venou*” OR “vessel*” OR “vasc*” OR “femo*” OR “ilii*” OR “aort*”
2. Surgical and procedural terms: “resect*” OR “recon*” OR “graft*” OR “excis*”
3. Additional anatomical or oncologic terms: “cytored*” OR “pelvic*” OR “exent*” OR “clearance*” OR “sidewall*” OR “lateral*”

The search was structured as: (vascular anatomy terms) AND (surgical/procedural terms) AND (additional terms). The MEDLINE search returned 1435 records. After removing duplicates, 848 unique records remained for screening.

Studies were selected using a staged review of titles and abstracts, followed by full‐text review. Titles and abstracts were screened independently by two reviewers (Y.O. and I.S.) to identify studies that potentially met the inclusion criteria. Full‐text articles were then assessed independently by the same reviewers for eligibility. Any discrepancies were resolved through discussion, with arbitration by senior authors where required.

The following data were extracted from selected articles onto an electronic proforma using MS Excel (CA, USA): author(s), year of publication, country of origin, journal of publication, journal impact factor, study design, number of patients included and number of centres involved. Patient demographics were also included as follows: age (median and range), sex ratio, ASA grade. Information about the pelvic tumour was extracted as follows: primary cancer type and histology, primary vs. recurrent cancer, TNM stage, EMVI (if colorectal in origin), resection intent (curative vs. palliative), utilisation of neoadjuvant or adjuvant treatment. Where appropriate, authors were contacted to provide missing information. For consistency, the term ‘internal iliac artery (IIA)’ is used throughout this manuscript in place of the synonymous term ‘hypogastric artery’.

### 2.3 Risk of Bias Assessment

A methodological assessment of risk of bias was undertaken, using the checklist detailed by the Risk‐of‐Bias Assessment tool for Non‐randomised Studies (ROBINS‐1 V2) [10]. Risk of bias was assessed using the ROBINS‐1tool across seven domains: target group selection, confounding variables, measurement of intervention, blinding of outcome assessors, outcome assessment, incomplete outcome data and selective outcome reporting. The primary outcomes were considered. It was determined if these criteria exhibited high, low, or unclear risk of bias. Two independent reviewers (Y.O. and I.S.) assessed each included study and presented the risk of bias for. Any disagreements were settled by the senior authors.

### 2.4 Outcomes of Interest

#### 2.4.1 Primary Outcome Measures

There is currently no core outcome set for oncovascular pelvic cancer resection. The primary outcomes of interest, R0 resection rate and Clavien–Dindo Grade V (30‐day mortality), were recorded. Other complications denoted by the Lexicon Collaboration of UKPEN and ACPGBI Advanced Cancer Subcommittee [8], including wound infection, urinary leak, return to theatre, anastomotic leak, compartment syndrome, venous thromboembolism, flap complications, pelvic collections, graft infection, target vessel thrombosis and 30‐day readmission, were recorded where available and stratified as per Clavien–Dindo. Although a broad range of complication outcomes was predefined, heterogeneity in reporting across studies limited consistent extraction. Therefore, R0 resection, overall morbidity (Clavien–Dindo) and 30‐day mortality were prioritised as primary outcomes due to their consistent reporting. A minimum follow‐up period was not predefined; however, all included studies reported at least 30‐day postoperative outcomes, in line with the primary endpoints of this review. These criteria are supported by recently published international benchmark data in pelvic exenteration surgery, which identify R0 resection and 30‐day mortality as key outcome domains for quality assessment and highlight the use of standardised complication grading to facilitate meaningful centre‐to‐centre comparison [11].

#### 2.4.2 Secondary Outcome Measures

Oncological outcomes were recorded including recurrence rate, median survival and disease‐free survival, where available. Preoperative data collected included the extent of vascular involvement was identified before surgery. Operative details collected included surgical approach (open/laparoscopic/robotic), whether the oncovascular procedure was part of the index operation or staged, mean operative time and estimated blood loss. The non‐oncovascular intraoperative sequence was recorded per the Lexicon Collaboration of UKPEN and ACPGBI Advanced Cancer Subcommittee [8]. Oncovascular details, including vascular resection and reconstruction technique, were tabulated following the approach demonstrated by Peacock et al.'s to capture variation in intraoperative methodology [12]. Where a single operation involved resection or reconstruction at multiple arterial levels (e.g., aortic, common iliac and external iliac segments), this was counted as one oncovascular arterial procedure at patient level, rather than as separate procedures for each segment. The inclusion of patient‐reported quality of life outcomes was noted. Where available, long‐term outcomes, including recurrence, 5‐year survival, disease‐free survival and follow‐up duration (mean and range), were recorded.

### 2.5 Statistical Analysis

The papers examined within this systematic review did not uniformly report outcomes, thereby rendering direct comparison and statistical analysis ineffectual. Descriptive statistics were performed using MS Excel (Ca, USA) and where primary data was not available, ranges of results are quoted. Due to observed inter‐study heterogeneity, a meta‐analysis was not performed. Pooled averages were reported as median values. Where mean values with distribution data were reported, an estimate of the median was calculated using Luo et al.'s proposed estimator [13]. Pre‐1980s studies were excluded from statistical analysis to report more accurately modern oncovascular practice.

## 3 Results

**FIGURE 1:**
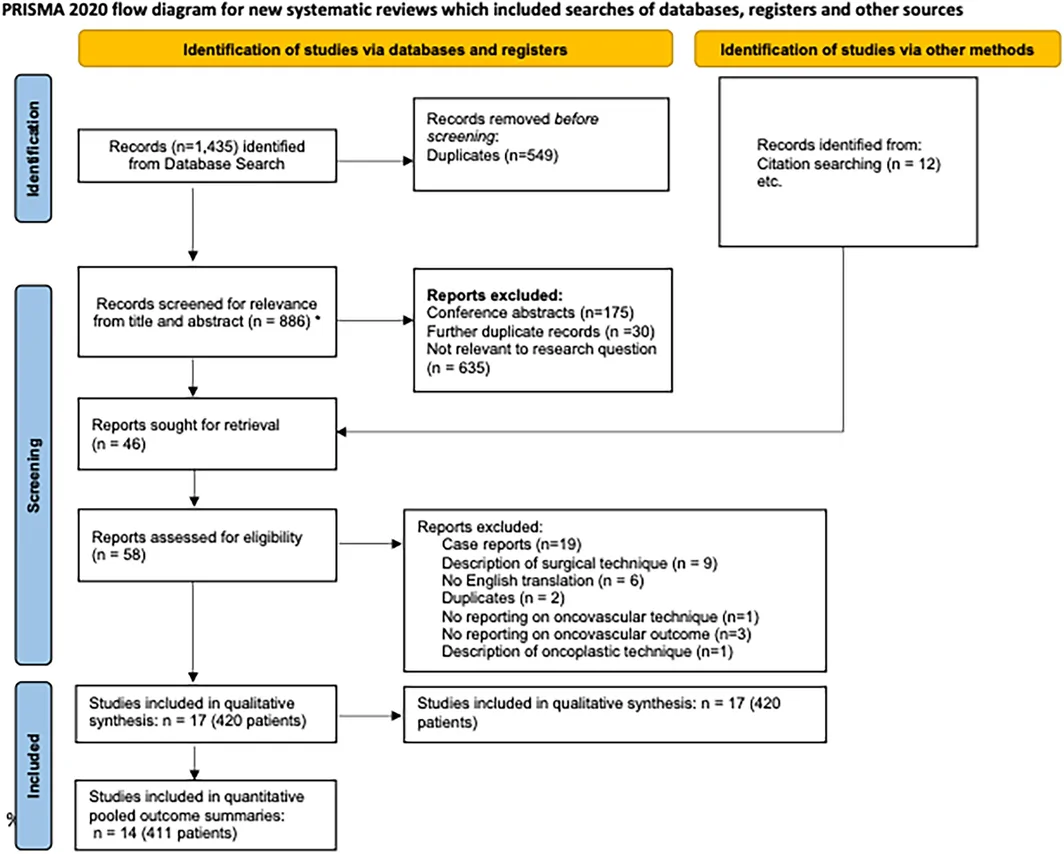
PRISMA flow diagram for the systematic review which details the searches of databases, registers, and other sources included in the systematic review. *Databases (*n* = 223), Ovid MEDLINE (*n* = 663) [9].

Initial literature search, following removal of duplicates, yielded 886 papers. A further 12 records were identified from citation searching (Figure 1). Following screening, 58 reports were retrieved and assessed for eligibility. Nineteen case reports were excluded. A further nine reports were removed as descriptions of surgical technique alone, without reporting of patient outcomes. Two reports were excluded due to a lack of reporting on the oncovascular technique used and a further report was excluded for not specifying outcomes within the oncovascular cohort.

**FIGURE 2:**
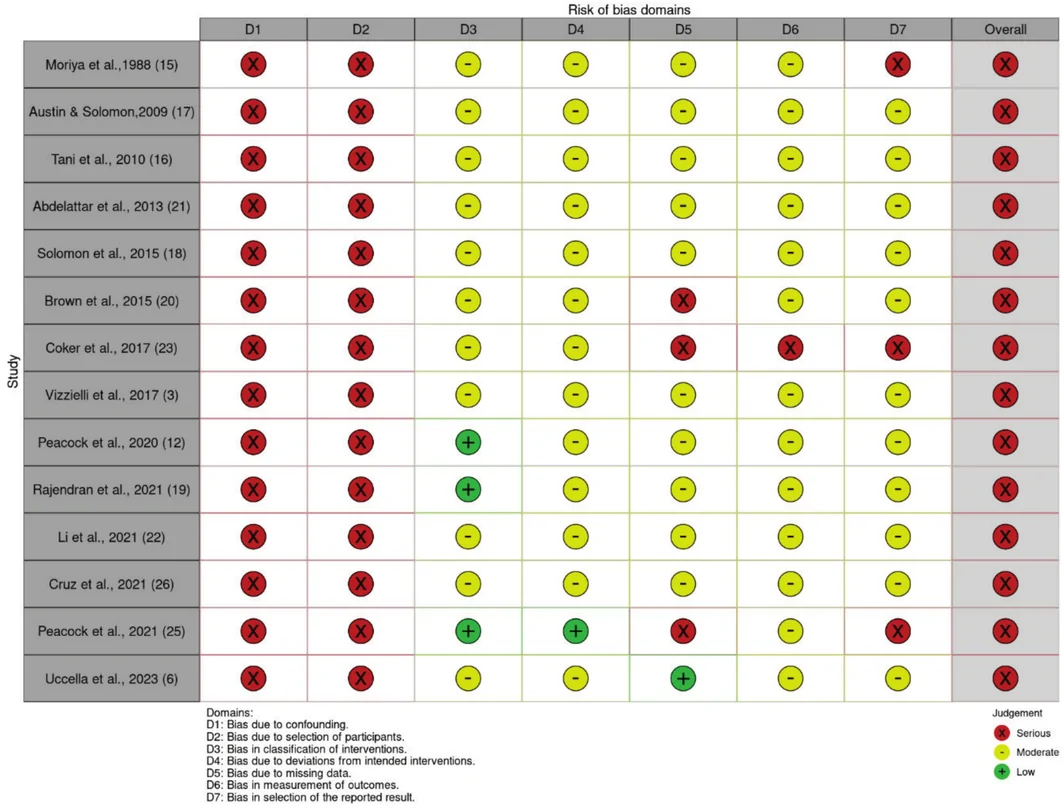
Risk of bias assessment using ROBINS‐1 V2. Selection of the reported result (D7) was judged based on omission of key expected outcomes, including margin status (R0 resection), postoperative complications, and mortality. Studies not reporting these outcomes were classified as serious risk of bias, while those with broadly complete but non‐pre‐specified reporting were classified as moderate [1, 3, 6, 12, 14, 15, 16, 17, 18, 19, 20, 21, 22, 23, 24, 25, 26]. Created using robvis tool [27].

After examination and exclusion, 17 studies were deemed suitable, including a total of 420 patients [1, 3, 6, 12, 14, 15, 16, 17, 18, 19, 20, 21, 22, 23, 24, 25, 26]. Three studies published before 1980 (*n* = 9) [2, 14, 24], did not report complications or survival in a way compatible with contemporary series. They were therefore included narratively only and excluded from quantitative synthesis due to non‐comparable historical surgical techniques. The contemporary cohort therefore comprised 411 patients across 14 studies published between 1980 and 2023. Bias was assessed and results displayed in Figure 2.

### 3.1 Primary Outcomes

Across all the contemporary studies R0 resection rate was reported, in which most procedures (*n* = 334) were performed with curative intent. Among the 334 procedures performed with curative intent, the R0 resection rate was 65.3% (218/334) and R1 was 29.3% (98/334). One study reported procedures achieving R0 and R1 margins and was not disaggregated [22].

Thirty‐day post‐operative complications were reported in all contemporary studies, although not all reported Clavien–Dindo grading. Although all contemporary studies reported postoperative complications, denominators varied according to available outcome reporting and these are preserved to prevent artificial inflation or suppression of rates. Minor complications (Clavien–Dindo I–II) were reported in 399 patients and occurred in 227 cases (56.9%). Major complications (Clavien–Dindo III–IV) were reported in 382 patients, among whom 109 (28.5%) experienced a severe event. A return to theatre occurred in 96 of 374 cases (25.7%) and graft‐related complications in 34 of 368 cases (9.2%). Across the contemporary cohort, 30‐day mortality (Clavien–Dindo grade V) was 1.9% (8/411). These outcomes highlight the substantial morbidity associated with oncovascular pelvic exenteration, even within contemporary practice. The most frequently reported complication was wound infection/abscess formation, occurring in 10% (43/420) of reported cases, consistent with the Lexicon Collaboration definitions.

### 3.2 Patient Demographics

Amongst the contemporary cohort, sex was reported amongst all 411 contemporary patients; with a gender split of 50.1% male (206/411) and 49.9% (205/411) female. Three patients with unspecified sex all belonged to pre‐1980 studies and were excluded from quantitative analyses [1]. Of the contemporary cohort, the median age of patients was 55.5 years (range 46–73 years). ASA grade was only reported in two of the papers included in the review [3, 23].

Over half of the contemporary patient cohort (58.6%, *n* = 241) had colorectal cancer and 10.0% (*n* = 41) had gynaecological malignancies. Gynaecological malignancies represented a minority of the cohort and included ovarian carcinoma, cervical cancer (including squamous cell carcinoma), uterine/endometrial adenocarcinoma and vaginal malignancies (including vaginal melanoma), reflecting the range of pelvic tumours requiring exenterative surgery with vascular involvement. Of the remaining patients, 4.6% (*n* = 19) had anal cancer and 5.6% (*n* = 24) had sarcoma. Recurrent disease accounted for 65% of cases (*n* = 269). Curative resection was reported in 81.3% (*n* = 334/411) across the 14 contemporary studies, with an overall R0 resection rate of 65.3% (218/334).

Around one third of patients were reported to have received some form of radiotherapy, either defined as previous radiotherapy (*n* = 120) or as part of a neoadjuvant regime for their recurrence (*n* = 22).

### 3.3 Operative Details

Across the 14 contemporary studies, operative approach and staging were variably reported. Five percent of patients (*n* = 21/411) across three studies underwent staged oncovascular procedures [14, 16, 25]. One study (*n* = 4) staged femoral‐femoral arterial and venous crossover with arteriovenous loop fistula first, followed by 4 weeks later radical pelvic exenteration [24]. Where reported, vascular resections formed part of pre‐planned exenterative procedures; none of the included studies described vascular excision undertaken purely as a salvage manoeuvre for intraoperative injury, although most did not explicitly classify excisions as ‘planned’ versus ‘unplanned’.

Robotic surgery was rare, with all cases within a single series (1.9%, *n* = 8) of laterally extended endopelvic resections [3]. Among studies reporting operative approach (*n* = 333), 254 procedures (76.3%) were performed open. Five contemporary studies reported the post‐operative histological findings [3, 6, 12, 16, 22]. Two reports detailed whether positive nodes were excised during treatment [16, 17]. Pooled median operating time was 520 min (interquartile range 450–615). Pooled median blood loss was 2000 mL (interquartile range 1690–4250) (Table S1).

### 3.4 Oncovascular Surgical Approach

Reporting of vessel dissection, excision and reconstruction varied considerably across studies. Three historical series published before 1980 [2, 14, 24] provided descriptive data only and did not consistently distinguish arterial from venous procedures or specify reconstruction techniques. These studies are therefore reported narratively but excluded from quantitative synthesis of oncovascular techniques. Of the contemporary papers, two papers did not distinguish between arterial and venous techniques in their reporting [17, 19]. Although aortic and inferior vena cava resections were described in a minority of patients, no study reported short‐ or long‐term outcomes stratified by aorto‐caval involvement, so the specific risk profile of these most complex resections remains undefined.

Among the 14 contemporary studies, excluding those that pooled arterial and venous involvement into a single category, a total of 148 arterial dissections and 124 venous excisions were reported. For the purposes of this review, arterial dissection denotes tumour being dissected off and freeing a preserved artery, whereas venous excision refers to segmental resection of the involved vein with or without reconstruction. Removing non‐attributable artery/vein pooled entries ensures that only clearly documented vessel types are included in the analysis of oncovascular involvement, although they are included in Table 2 for reference. Arterial dissection was reported in 148 cases, representing 36% of the contemporary cohort (148/411). Arterial reconstruction was reported explicitly in 96 cases, corresponding to 65% of all arterial dissections (96/148) and 23% of all contemporary procedures (96/411). Reporting of arterial reconstruction technique and graft material varied considerably across studies, and ratios are presented only where these details were explicitly described. Among procedures where the reconstruction technique was specified (*n* = 45), interposition grafting was the most frequently reported method (22/45; 49%), followed by femoral–femoral crossover bypass (8/45; 18%). Other described techniques included patch repair, primary repair, end‐to‐end anastomosis, arteriovenous loop fistula formation, and interposition graft, each reported infrequently. End‐to‐end reconstructions restore continuity between proximal and distal vessel ends (through an interposition graft if bridging a gap or primary repair if no resection), whereas bifurcated graft reconstructions provide inflow to two distal branches from a single proximal source. Graft material was specified in 62 arterial reconstructions. Synthetic grafts, including polytetrafluoroethylene (PTFE), Dacron (polyester) and other artificial conduits, were used in 44 cases (44/62; 71%). Autologous conduits were used in 9 cases (9/62; 15%), cryopreserved arterial allografts in 7 cases (7/62; 11%), and bovine pericardium in 2 cases (2/62; 3%). Cases with incomplete reporting of graft material were excluded from these ratios.

Venous involvement was also notable, with 124 clearly attributable venous excisions reported across contemporary series, representing 30.1% of the total cohort (124/411). The external and common iliac veins were the most frequently affected structures, while inferior vena cava involvement was identified in nine cases, corresponding to 7.3% of all venous excisions (9/124) and 2% of the entire study population (9/411). Overall, 49 venous procedures (39.5%, 49/124) proceeded without reconstruction. Reporting of venous reconstruction was variable across studies, and ratios are presented only where the technique or material was explicitly described. Among procedures where reconstruction technique was specified (*n* = 45), interposition grafting was the most common (24/45; 53%), followed by primary repair (8/45; 18%), patch repair (6/45; 13%), femoral–femoral crossover grafting (6/45; 13%), and spleno‐renal shunting (1/45; 2%). Reconstruction materials were specified in 35 procedures: autologous vein in 22 cases (22/35; 63%), PTFE grafts in 12 cases (12/35; 34%), and bovine pericardium in one case (1/35; 3%).

A comprehensive comparison of oncovascular surgical approaches, including those where reporting is mixed, is summarised in Table 2.

### 3.5 Oncological and Patient‐Reported Outcomes

Survival outside of the 30‐day post‐operative window was not reported uniformly. Of the five contemporary papers which reported median overall survival, the pooled median (range) was 41 months (range 34–57.6). Median disease‐free survival was reported in seven contemporary papers, of which the pooled median was 27 (range 8–43.2) months.

Long‐term survival also varied substantially. Recurrence was reported in nine studies, with 36 events among 236 patients for whom recurrence data were available (15.3%). Reported recurrence rates varied widely between studies, reflecting heterogeneity in tumour biology, disease stage, and duration of follow‐up. Four papers reported the site of metastasis identified during the follow up period [3, 15, 16, 17]. Five‐year survival rate was reported in four contemporary papers, and ranged from 35% to 70%, with a pooled median of 51% [16, 18, 19, 21] (Table 1).

**TABLE 1:** Summary of reported outcome measures in the contemporary studies included in quantitative synthesis.

Study	Patients (*n*)	Clavien–Dindo grade I–II (*n*)	Clavien–Dindo grade III–IV (*n*)	R0 (*n*)	Median follow‐up time (months [range])	Median survival (months [range])	Return to theatre (*n*)	30‐day mortality (*n*)	Median disease‐free survival (months [range])	5‐year survival rate (%, [range])
Moriya et al. [15]	8	1 (12.5%)	2 (25%)	—	—	36 (9–93)	—	0	—	—
Austin and Solomon [17]	36	6 (16.7%)	6 (16.7%)	19	19.2 (4–140)	—	3 (8.3%)	0	30***	—
Tani et al. [16]	7	1 (14.2%)	0	7	—	57.6 (25–112)	0	0	43.2	70
Abdelattar et al. [21]	12	8 (66.7%)	1 (8.3%)	7	—	—	1 (8.3%)	0	—	55
Solomon et al. [18]	21	8 (38.1%)	9 (42.9%)	8	38 (0–222)	41^a^	5 (23.8%)	0	27****	35
Brown et al. [20]	200	158 (79%)	55 (27.5%)	126	—	34^a^	67 (33.5%)	0	26	—
Coker et al. [23]	25	17 (68%)	—	—	—	—	—	1 (4%)	—	—
Vizzielli et al. [3]	8	—	4 (50%)	6	19 (9–28)	—	2 (25%)	0	28 (7–64)	—
Peacock et al. [12]	11	3 (27.3%)	1 (9.1%)	9	22 (4–58)	—	1 (9.1%)	0	—	—
Rajendran et al. [19]	50	17 (34%)	27 (54%)	30	—	41 (30–52)	13 (26%)	2 (4%)	—	47
Li et al. [22]	15	2 (13.3%)	0	15 (R1 included)	20.8*** (4–77)	—	0	0	8*****	—
Cruz et al. [26]	9	5 (55.6%)	3 (33.3%)	—	23 (3–103)	—	3 (33.3%)	0	—	—
Peacock et al. [25]	4	—	—	4	—	—	—	—	—	—
Uccella et al. [6]	5	1 (20%)	1 (20%)	2	5.5 (5–25)	—	1 (20%)	1 (20%)	15.5 (5–25)	—
	411	227/399 (56.9%)	109/382 (28.5%)	233	20.0 (5.5–57.6)	41 (34–57.6)	96/374 (25.7%)	8/411 (1.9%)	27 (8–43.2)	51 (35–70)

*Note:* The table details the number of cases for each study in which complications were classified according to Clavien–Dindo Grades I/II (minor complications) and III/IV (major complications), as well as the number of 30‐day postoperative deaths. Number of R0 resections (histologically clear margins), median follow‐up, overall and disease‐free survival (months), and 5‐year survival where reported. Data demonstrate interstudy heterogeneity, highlighting the need for standardised reporting. Where data is missing, this is denoted by a (—) [3, 6, 12, 15, 16, 17, 18, 19, 20, 21, 22, 23, 25, 26].

*** Mean value.

**** Patients only included in value if R0 resection achieved.

***** Patients who died only included.

^a^ Range not stated.

Patient‐reported outcomes were only available in two studies. One series assessing postoperative quality of life following pre‐emptive internal iliac vein ligation reported meaningful recovery by 3–6 months, while another using the Barthel Index demonstrated restored functional independence in the majority of patients following extended lateral pelvic resections. However, the sparse and inconsistent nature of reporting prevents reliable synthesis of patient‐reported outcome measures, and these findings should be interpreted cautiously.

## 4 Discussion

Irrespective of oncovascular involvement, pelvic exenteration is an ultra‐radical procedure which, in contrast to its historic palliative use, is reserved for patients who are most likely to gain long‐term survival benefit. The PelvEx Collaborative reviewed 273 pelvic exenteration procedures amongst colorectal cancers and reported an R0 resection rate of 84.2% [28], compared to 65.3% (218/334) across contemporary oncovascular studies. Although there are limitations in this comparison due to heterogeneous tumour cohorts, this suggests that vascular invasion of tumours reflects advanced disease and in which achieving clear margins is more challenging. R0 resection remains a significant prognostic indicator for long‐term survival, and the need for oncovascular resection may itself be a surrogate marker of biologically aggressive or more locally advanced disease, with greater risk of recurrence and metastasis [28].

It is important to note that outcomes from the PelvEx Collaborative reflect a heterogeneous international cohort encompassing multiple centres with variable volume and experience. Recent attempts to define evidence‐based benchmarks for pelvic exenteration most notably by Brown et al. [11] demonstrate that R0 resection and 30‐day mortality rates in specialist centres are in keeping with or exceed those in broader collaborative datasets, underscoring the impact of institutional experience and case volume on oncovascular outcomes. While the pooled R0 rate in the present systematic review is broadly comparable to the PelvEx cohort, it remains below that reported in benchmark series from high‐volume specialist centres. Oncovascular pelvic exenteration requires a highly specialised multidisciplinary approach, and these procedures are typically undertaken in high‐volume centres with expertise in complex pelvic oncology and vascular surgery, with close intraoperative collaboration between surgical teams. Given the risk of major haemorrhage, institutional preparedness, including access to blood products and established massive transfusion protocols, is essential to support safe operative delivery.

Tumour resection involving major blood vessels is anticipated with longer operating times, greater intra‐operative blood loss, and higher post‐operative complication rates, given the added complexity of vascular procedures and the disruption to pelvic and lower limb perfusion. In our review, operating times were comparable to those reported in the PelvEx study (528 min; IQR 390–648) [28]. However, intra‐operative blood loss was substantially higher (median 2000 mL; IQR 1690–4250) compared with 1100 mL (IQR 900–2450) in the PelvEx cohort. Despite this, increased blood loss did not translate into a higher rate of major complications: the return‐to‐theatre rate was 25.7%, consistent with the PELVEX study, which reported a major complication rate of 22% [28]. These findings suggest that although oncovascular procedures increase operative complexity and technical demands, this does not necessarily result in disproportionate postoperative morbidity. Reporting of vascular‐specific outcomes, particularly short‐ and long‐term graft patency, was inconsistent across studies and could not be meaningfully synthesised. Standardised reporting of vascular outcomes will be essential in future studies to better evaluate the durability of oncovascular techniques.

Caution must be exercised when comparing outcomes from the PelvEx audit with those of the present cohort, given the well‐documented heterogeneity in reporting standards and case mix across exenteration series [29]. While the development of the *Pelvic Exenteration Lexicon* represents a major step towards standardised nomenclature, it does not yet define oncovascular pelvic exenteration as a distinct subgroup, instead including these within the broader “high‐complexity” category, where resection of major vessels, sciatic nerves or bone is undertaken [8]. Recent prospective data from The Royal Prince Alfred Hospital in Sydney, Australia [11], further delineate outcomes in this high‐complexity cohort, reporting longer operative times (median 550 min), increased transfusion requirements, and higher rates of reoperation compared with conventional exenteration, yet with comparable short‐term mortality and superior long‐term survival outcomes in selected patients. These contemporary findings reinforce that, in expert centres, high‐complexity or oncovascular pelvic exenteration can be performed safely, provided appropriate patient selection and multidisciplinary support are in place [28].

Despite comprehensive outcome reporting, the certainty of evidence is low, based on GRADE criteria [29]. All included studies were observational (retrospective cohorts or case series), thereby inherently limiting confidence in effect estimates. Key factors contributing to low certainty include a high risk in patient selection in 68% of studies (ROBINS‐1) (Figure 1) with wide confidence intervals in survival outcomes 41 months (range 34–57.6) inconsistency; with R0 resection rates varying widely across institutions, and indirectness with data pooled from mixed tumour types—colorectal (57%), gynaecological (10%), and sarcoma (6%). Furthermore, the inclusion of multiple tumour types, including colorectal, gynaecological malignancies, and sarcoma, introduces heterogeneity, thereby influencing reported outcomes; however, this reflects real‐world practice where oncovascular techniques are applied across tumour subtypes in anatomically comparable disease. These limitations warrant cautious interpretation of reported survival and complication outcomes.

Our findings likely overestimate survival outcomes due to underreporting of unsuccessful cases. Although study heterogeneity precluded funnel plot analysis, several factors suggest a bias toward positive results. Notably, all included studies originated from high‐volume referral centres, and no published reports described complete resection failure. However, grey literature searches identified unpublished cases of catastrophic graft failure from non‐specialist centres, highlighting the likelihood of selective reporting and publication bias [6].

This review establishes there is great variability in the intraoperative vascular techniques deployed in this cohort of patients. The inconsistent reporting surrounding vascular techniques makes statistical analysis of the data difficult. This review adopted the systematic approach of Peacock et al. in its reporting, but as demonstrated in Table 2, not all papers provided sufficient detail to achieve this uniformly. Cases often involve contemporaneous tumour resection and oncovascular reconstruction; however, staged operations have been established, wherein the oncovascular reconstruction is performed prior to tumour resection [25]. Staged procedures are established as safe and enable greater control over reconstruction; however, there is an associated increase in anaesthetic risk for the patient, and resource consumption of the unit, and given the small sample size analysed, it is difficult to suggest this demonstrated any reduction in morbidity through reduced haemorrhage [25]. Other potential techniques include pre‐operative embolisation, although current thoughts are this may increase intraoperative venous haemorrhage due to the formation of collateral vessels [25]. Reconstruction of the aortoiliac system is achieved through bypass grafting, although there is variability in the use of synthetic or autologous grafts, including reports of great saphenous vein grafting [7, 30]. Some reports exclusively describe the use of synthetic artery grafts [16, 22], whereas other studies use an individualised approach based on available allograft sites [3, 6, 19, 20, 26, 30]. The management of the pelvic venous drainage system is dependent on patency of the vessels. When there is associated venous thrombosis or chronic occlusion of the iliac vein, the vessel is ligated without reconstruction, with the view that the collateral venous circulation is sufficient [7, 19]. Adequate collateral venous drainage may be noted on pre‐operative imaging, enabling the sacrifice of major veins in the tumour resection without further reconstruction [3, 6, 23]. Reconstruction of the venous tracts is demonstrated in the literature, although choice of graft, and whether to form an arteriovenous fistula to ensure blood flow, remain controversial [7, 16, 25]. The use of oncovascular techniques also elicits consideration between managing anticoagulation and risk of haemorrhage; however, this avenue is underreported and thereby was not analysed. To systematically evaluate which oncovascular techniques are associated with optimal outcomes, a standardised and comprehensive framework for reporting oncovascular methods is essential.

**TABLE 2:** Vascular dissection and reconstructions as reported in the 17 papers included in the systematic review.

Study	Arterial dissection	Arterial reconstruction	Venous excision	Venous reconstruction
*Brunschwig and Walsh [24]	—	—	EIV/IIA—2 CIV + EIV + IIA—2	No reconstruction—4
*Engler and Mcinnes [14]	CIA + EIA—1 EIA—1	Autologous graft—1 Ivalon Teflon graft—1	IVC + CIV + EIV—1 EIV—1	No reconstruction—2
*Barber and Brunschwig [1]	EIA—3	Not stated—3	—	—
Moriya et al. [15]	IIA—8	No reconstruction—8	IIV—8	No reconstruction—8
Austin and Solomon [17]	Aorta—1 CIA—8 EIA—8 IIA—36	Not stated—53	IVC—1	Not specified
Tani et al. [16]	Aorta—2 CIA + aorta—5	Synthetic—7	IVC + Renal—1 Partial IVC—1	Spleno‐renal shunt—1 No reconstruction—1
Abdelattar et al. [21]	Aorta—3 CIA—5 EIA—3	Reconstructed but not specified—11	EIV—1	Reconstructed but not specified—1
*Solomon et al. [18]	CI or EI A/V—29 Reconstruction/repair—23. No reconstruction—6			
*Brown et al. [20]	Vein—10 Artery—3 Both artery and vein—8 Autologous reconstruction—16 Synthetic (PTFE graft) reconstruction—5 Patch repair—2 Primary repair—1 No reconstruction—5			
Coker et al. [23]	—	—	IIV—25	No reconstruction—25
Vizzielli et al. [3]	EIA—1 CIA—1	Fem‐fem cross over—1 Primary repair—1	CIV + EIV—1	No reconstruction—1
Peacock et al. [12]	CIA + EIA + IIA—4 EIA + IIA—3 EIA—2 CIA + EIA + IIA + Aorta—1	Fem‐fem cross over—7 Interposition graft—1 Both Fem‐fem and interposition—1 Autologous graft—1 Dacron graft—9	CIV + EIV + IIV—6 EIV + IIV—3	Fem‐fem crossover—5 Interposition—4 Autologous—1 PTFE—8
Rajendran et al. [19]	CIA + EIA—20 CIA—3 EIA—2	Interposition—20 Primary repair—1 End to end reconstruction—2 No reconstruction—2 Autologous—7 PTFE graft—11 Bovine pericardium—2	CIV + EIV—24 CIV—9 EIV—12	Interposition—20 Fem‐fem crossover—1 Patch—5 Primary repair—8 No reconstruction—11 Autologous—21 Bovine pericardium—1
Li et al. [22]	Unilateral IIA—10 Bilateral IIA—5	All bifurcated graft artificial arterial reconstruction—15	IVC partial resection—2 IVC partial transection—1 CIV ligation—3 IIV ligation—1	—
Cruz et al. [26]	Aorta—2 CIA + EIA—3 CIA—1 Superficial femoral—1	Cryopreserved allograft—7 Dacron graft—2	—	—
Peacock et al. [25]	CIA + EIA—4	Fem‐fem crossover—4 Arteriovenous loop fistula—4	CIV + EIV—4	PTFE graft—4
Uccella et al. [6]	CIA—1 EIA—2	End‐to‐end anastomosis—1 Autologous—1 No reconstruction—1	EIV—1 IVC + CIV—2	Not reconstructed—3

*Note:* The following table utilised the methodology in reporting of oncovascular technique demonstrated by Peacock et al. [12]. A key of abbreviations is demonstrated below the table. Where data is missing, this is denoted by a (—) [1, 3, 6, 12, 14, 15, 16, 17, 18, 19, 20, 21, 22, 23, 24, 25, 26]. End‐to‐end—anastomosis between two vessel ends (with or without interposition graft). Bifurcated graft reconstruction—graft configuration in which a single inflow is anastomosed to two distal branches forming a Y‐shaped construct. Papers marked with an asterisk (*) do not distinguish between arterial and venous resection and reconstruction. The shaded articles were included for contextual purposes but were not included in the analysis because of their historical nature. The authors judged that these studies are not representative of contemporary surgical practice.

Abbreviations: CIA—common iliac artery; CIV—common iliac vein; EIA—external iliac artery; EIV—external iliac vein; IIA—internal iliac artery; IIV—internal iliac vein; IVC—inferior vena cava.

Radiotherapy is used within the treatment of sarcoma [19, 21, 25], gynaecological [1, 3, 11, 13, 17], and colorectal malignancies [11, 17, 18]. This establishes a growing population of patients presenting with recurrent or persistent disease in what is often referred to as a ‘hostile pelvis’, an anatomical field altered by prior irradiation, leading to fibrosis, inflammation, and distorted tissue planes. These changes correspond with higher rates of breached planes, which may contribute to the 25.7% return‐to‐theatre rate observed in our cohort.

Seven of the studies involved the treatment of rectal cancer. The widespread adoption of total neoadjuvant therapy (TNT) for locally advanced rectal cancer presents emerging challenges for oncovascular surgery. TNT improves pathological complete response rates (36% vs. 21%) and reduces distant metastases [31]. However, intensified regimens such as FOLFIRINOX combined with long‐course chemoradiation profoundly alter pelvic tissue architecture, as post‐TNT MRI studies report a 42% increase in perirectal fibrosis compared with pre‐operative chemoradiation alone, characterised by vessel encasement, reduced tissue elasticity, and impaired perfusion [32]. Additionally, delayed surgery to accommodate chemotherapy exacerbates fibrosis and vascular compromise [33], increasing risks such as iliac artery calcification, collateral loss, and graft infection, which may in part explain the graft‐related complication rate of 9.2% reported here.

Looking ahead, this complexity is likely to intensify with the increasing adoption of pelvic re‐irradiation protocols, as explored in GRECCAR 14 [34] and PelvEx II [35]; such approaches, while promising, are expected to further increase fibrosis and obliteration of tissue planes, presenting significant technical challenges for future oncovascular resections.

Future patient selection may benefit from integrating pelvimetric indices, fibrosis biomarkers (e.g., TGF‐β1), and dynamic perfusion MRI to identify high‐risk cases [36, 37]. Proactive venous mapping, currently underreported, should become standard practice in patients previously treated with radiotherapy who subsequently undergo oncovascular resection.

Based on our findings, we suggest several considerations to support clinical decision‐making in oncovascular resections. During patient counselling, it is important to highlight the 25.7% return‐to‐theatre rate for major complications and the substantial risk of recurrence (15.3% in this systematic review), even when R0 resection is achieved. At the institutional level, a minimum annual caseload of oncovascular resections may help maintain surgical expertise, and participation in international registries such as the PelvEx Collaborative [28] could facilitate benchmarking, quality improvement, and data transparency.

## 5 Conclusion

Oncovascular pelvic exenteration represents a vital surgical strategy for select patients with advanced pelvic malignancies involving the aorto‐iliac axis. While this approach is associated with a lower R0 resection rate compared with the recent PelvEx series (65.3% vs. 84.2%) and may consequently confer poorer long‐term survival, it nonetheless offers a potentially curative option for patients who would otherwise be deemed inoperable. Importantly, perioperative mortality remains acceptable, and the overall complication profile is comparable to conventional pelvic exenteration, despite significantly increased intra‐operative blood loss. Careful patient selection and comprehensive pre‐operative counselling are essential, given the substantial morbidity associated with these procedures.

The current evidence base is characterised by retrospective designs, heterogeneous reporting standards, and institutional variability in surgical technique, graft choice, and perioperative management. Taken together, these data suggest that oncovascular pelvic exenteration has historically been associated with higher re‐operation rates and seemingly worse outcomes than conventional exenteration, with more than one in five patients requiring a return to theatre and only around two‐thirds achieving R0 resection. Much of this excess morbidity and lower margin positivity likely reflects the pioneering phase of oncovascular practice, with many included series predating contemporary imaging, neoadjuvant protocols, and standardised exenteration pathways. Encouragingly, R0 rates appear higher and practice more standardised in the most recent series, supporting the view that oncovascular exenteration is now moving from an experimental phase to a quality‐improvement phase focused on better patient selection, centralisation, and rigorous audit and benchmarking. The priority should therefore be to drive R0 rates up and complication rates down through structured quality‐improvement initiatives, rather than accepting comparatively poor outcomes as an inevitable consequence of tumour location or complexity.

As surgical boundaries continue to expand in the context of increasing adoption of TNT, the deployment of complex operations in a hostile pelvis is likely to increase, and oncovascular techniques will have broader applications. To ensure optimal safety and efficacy, minimum institutional caseloads, multidisciplinary collaboration, standardised terminology, and registry participation are strongly recommended. Current outcomes likely represent the early, pioneering phase of oncovascular practice; future efforts must focus on centralisation, benchmarking, and systematic quality improvement to narrow the gap with conventional exenteration while preserving access to potentially curative surgery. Ultimately, oncovascular pelvic exenteration should be undertaken in high‐volume specialist centres within a framework that prioritises evidence‐based, patient‐centred care.

## Author Contributions

J.R.B. and J.G. conceived the study The search‐identified abstracts and those from additional sources were screened independently by Y.O. and I.S. to identify studies that potentially met the inclusion criteria outlined previously. Discordance was settled by arbitration by the senior authors. Y.O. and J.R.B. drafted the initial manuscript. J.W., J.G., K.G.B., P.A.S. and J.R.B. reviewed the final manuscript.

## Funding

P.A.S. is funded by The Christie Charity, in partnership with NIHR Manchester Biomedical Research Centre. J.R.B. is funded through an NIHR Academic Clinical Fellowship. The views expressed are those of the author(s) and not necessarily those of the NIHR or the Department of Health and Social Care.

## Ethics Statement

The authors have nothing to report.

## Conflicts of Interest

The authors declare no conflicts of interest.

## Supporting information


**Table S1:** Summary of intraoperative parameters and major complications reported across studies included in this systematic review. For each paper, the table displays the mean operating time and mean intraoperative blood loss, providing insight into the technical demands and complexity of pelvic exenteration procedures. Additionally, the table enumerates the number of patients who experienced specific complications, as defined by the 2024 PelvEx audit criteria. These complications include anastomotic leak, wound infection, urinary tract injury, deep vein thrombosis, pulmonary embolism and major vascular injury.
**Table S2:** Search Strategy. The following search strategy was used in performing the systematic search.

## Data Availability

The data that support the findings of this study are available from the corresponding author upon reasonable request.
