# Supplementary material for: Oncovascular Resection in Pelvic Exenteration: A Systematic Review
Source: ANZ J Surg. 2026 Jul 7;96(9):2308–20. doi: 10.1111/ans.70824 (PMC13602428; doi:10.1111/ans.70824)
Supplement: Supplementary file 1 [file ANS-96-2308-s001.docx]

**Oncovascular Resection in Advanced Pelvic Malignancy; A Systematic Review.**

**Supplementary Figures/Tables**

**Supplement Table 1.** Summary of intraoperative parameters and major complications reported across studies included in this systematic review. For each paper, the table displays the mean operating time and mean intraoperative blood loss, providing insight into the technical demands and complexity of pelvic exenteration procedures. Additionally, the table enumerates the number of patients who experienced specific complications, as defined by the 2024 PelvEx audit criteria. These complications include anastomotic leak, wound infection, urinary tract injury, deep vein thrombosis, pulmonary embolism, and major vascular injury.

|  | **Authors** | **Mean operating time (mins)**  **(SD)** | **Mean blood loss (ml)**  **(SD)** | **Wound infection** | **Urine leak** | **Arterial anastomotic leak** | **Bleeding requiring surgical intervention** | **Target arterial vessel thrombosis** | **Target venous thrombosis** | **Flap complications** | **Re-admission from 30 days** | **Graft infection** | **Compartment syndrome** |
| --- | --- | --- | --- | --- | --- | --- | --- | --- | --- | --- | --- | --- | --- |
| Brunschwig & Walsh, 1949 (24) | | - | - | 0 | 0 | 0 | 0 | 1 | 0 | 0 | 0 | 0 | 0 |
| Engler & McInnes, 1958 (14) | | - | - | 0 | 0 | 0 | 0 | 0 | 0 | 0 | 0 | 0 | 0 |
| Barber & Brunschwig, 1967 (1) | | - | - | 0 | 0 | 0 | 0 | 0 | 0 | 0 | 0 | 0 | 0 |
| Moriya et al., 1988 (15) | | 529 | 1675 | 2* | 1 | 0 | 0 | 0 | 0 | 0 | 0 | 0 | 0 |
| Austin & Solomon, 2009 (17) | | 540 (range 180–960) | - | 3 | 0 | 0 | 1 | 0 | 0 | 4 | 0 | 0 | 0 |
| Tani et al., 2021 (16) | | 356† | 1793 | 0 | 0 | 0 | 0 | 0 | 0 | 0 | 0 | 0 | 0 |
| Abdelsattar et al., 2013 (21) | | - | - | 0 | 0 | 0 | 0 | 0 | 0 | 0 | 0 | 0 | 0 |
| Solomon et al., 2015 (18) | | 615 (165–1200)† | 3500 (0–25,000) | 24 | 28 | 0 | 6 | 1 | 2 | 5 | - | 0 | 2 |
| Brown et al., 2015 (20) | | 631 | 6800 (1000–16,000) | 5 | 3 | 0 | 1 | 1 | 5 | 3 | - | 1 | 1 |
| Coker et al., 2017 (23) | | 510 | 5500 | 2 | 0 | 0 | 0 | 0 | 0 | 0 | 0 | 0 | 0 |
| Vizzielli et al., 2017 (3) | | 380 (165–499) | 1125 (300–3750) | 4** | 0 | 0 | 1 | 0 | 0 | 0 | 0 | 0 | 2 |
| Peacock et al., 2020 (12) | | 510 (330–960) | 2000 (500–3000) | 1 | 0 | 0 | 0 | 1 | 0 | 0 | 0 | 0 | 0 |
| Rajendran et al., 2021 (19) | | 678 (222–2166) | 6000 (0–16,000) | 5 | 1 | 0 | 6 | 1 | 0 | 0 | 0 | 0 | 2 |
| Li et al., 2022 (22) | | - | 1700 (500–5200) | 0 | 1 | 0 | 1 | 1*** | - | - | - | 0 | 0 |
| Cruz et al., 2021 (26) | | 520 (120–690) | 2000 (75–15,000) | 2*** | 0 | 0 | 1 | 1**** | 0 | 0 | - | 0 | 0 |
| Peacock et al., 2021 (25) | | - | - | 0 | 0 | 0 | 0 | 0 | 0 | 0 | 0 | 0 | 0 |
| Uccella et al., 2023 (6) | | 450 (361–564) | 1500 (400–4000) | 1 | 0 | 0 | 0 | 0 | 1 | 0 | 0 | 0 | 1 |

† median and range

*Both had abscess at primary site

**3 pelvic collection, 1 wound infection

***1 pelvic collection, 1 fascial dehiscence

****DVT

**Supplement Table 2 – Search Strategy.** The following search strategy was used in performing the systematic search.

| **#** | **Query** | **Results from 1 Nov 2024** |
| --- | --- | --- |
| 1 | "arter*" .m_titl. | 1,077,936 |
| 2 | "vein*" .m_titl. | 192,541 |
| 3 | "venou*" .m_titl. | 217,541 |
| 4 | "vessel*" .m_titl. | 118,949 |
| 5 | "vasc*" .m_titl. | 519,639 |
| 6 | "femo*" .m_titl. | 135,093 |
| 7 | "ili*" .m_titl. | 47,719 |
| 8 | "aort*" .m_titl. | 421,325 |
| 9 | "resect*" .m_titl. | 273,602 |
| 10 | "recon*" .m_titl. | 353,004 |
| 11 | "graft*" .m_titl. | 301,325 |
| 12 | "excis*" .m_titl. | 67,990 |
| 13 | "cytored*" .m_titl. | 11,666 |
| 14 | "pelvic*" .m_titl. | 114,536 |
| 15 | "exent*" .m_titl. | 4,511 |
| 16 | "clearance*" .m_titl. | 65,081 |
| 17 | "sidewall*" .m_titl. | 970 |
| 18 | "lateral*" .m_titl. | 185,862 |
| 19 | 1 or 2 or 3 or 4 or 5 or 6 or 7 or 8 | 2,525,742 |
| 20 | 9 or 10 or 11 or 12 | 962,988 |
| 21 | 13 or 14 or 15 or 16 or 17 or 18 | 377,739 |
| 22 | 19 and 20 and 21 | 1,435 |
| 23 | remove duplicates from 22 | 848 |
